# Cephalometric predictors of hypernasality and nasal air emission

**DOI:** 10.1590/1678-7757-2021-0320

**Published:** 2021-10-11

**Authors:** María Alicia DENEGRI, Patrick Pedreira SILVA, Maria Inês PEGORARO-KROOK, Terumi Okada OZAWA, Renato Yassutaka Faria YAEDU, Jeniffer de Cássia Rillo DUTKA

**Affiliations:** 1 Universidade de São Paulo Hospital de Reabilitação de Anomalias Craniofaciais Programa de Pós-Doutorado BauruSP Brasil Universidade de São Paulo, Hospital de Reabilitação de Anomalias Craniofaciais, Programa de Pós-Doutorado, Bauru, SP, Brasil.; 2 Universidad Nacional de Cuyo Facultad de Odontologia Mendoza Argentina Universidad Nacional de Cuyo. Facultad de Odontologia. Cátedra de Cirugía Bucomaxilofacial, Mendoza, Argentina.; 3 Universidade de São Paulo Hospital de Reabilitação de Anomalias Craniofaciais Programa de Pós-Graduação em Ciências da Reabilitação BauruSP Brasil Universidade de São Paulo, Hospital de Reabilitação de Anomalias Craniofaciais, Bauru-SP, Programa de Pós-Graduação em Ciências da Reabilitação, Bauru, SP, Brasil.; 4 Universidade de São Paulo Faculdade de Odontologia de Bauru Departamento de Fonoaudiologia BauruSP Brasil Universidade de São Paulo, Faculdade de Odontologia de Bauru, Departamento de Fonoaudiologia, Bauru, SP, Brasil.; 5 Universidade de São Paulo Faculdade de Odontologia de Bauru Programa de Pós-Graduação em Fonoaudiologia BauruSP Brasil Universidade de São Paulo, Faculdade de Odontologia de Bauru, Programa de Pós-Graduação em Fonoaudiologia, Bauru, SP, Brasil.; 6 Universidade de São Paulo Hospital de Reabilitação de Anomalias Craniofaciais BauruSP Brasil Universidade de São Paulo, Hospital de Reabilitação de Anomalias Craniofaciais, Divisão de Odontologia, Setor de Ortodontia, Bauru, SP, Brasil.; 7 Universidade de São Paulo Faculdade de Odontologia de Bauru Departamento de Cirurgia, Estomatologia BauruSP Brasil Universidade de São Paulo, Faculdade de Odontologia de Bauru, Departamento de Cirurgia, Estomatologia, Patologia e Radiologia, Bauru, SP, Brasil.

**Keywords:** Cleft palate, Cephalometry, Soft palate, Speech disorders, Velopharyngeal insufficiency

## Abstract

During times of increasingly recognized importance of interprofessional practices, professionals in Medicine, Dentistry, and Speech Pathology areas cooperate to optimize treatment of velopharyngeal dysfunction (VPD), after primary palatoplasty for correction of cleft palate.

**Objective:**

Our study aims to compare velar length, velar thickness, and depth of the nasopharynx of patients with unilateral cleft lip and palate (UCLP) with the presence, or absence, of hypernasality and nasal air emission; and to verify if the depth:length ratio, between nasopharynx and velum, would be predictive of consistent hypernasality and nasal air emission (speech signs of VPD).

**Methodology:**

Cephalometric radiographs and outcome of speech assessment were obtained from 429 individuals, between 6 and 9 years of age, with repaired unilateral cleft lip and palate. Velar length, velar thickness, depth of the nasopharynx, depth:length ratio, scores of hypernasality, and scores of nasal air emission were studied and compared; grouping the radiographs according to presence or absence of hypernasality and nasal air emission.

**Results:**

For the group with speech signs of velopharyngeal dysfunction (those with consistent hypernasality and nasal air emission), the velums were shorter and thinner; the nasopharynx was deeper and the depth:length ratio was larger than the group without hypernasality and nasal air emission. Velar length was significantly shorter in individuals with consistent hypernasality and nasal air emission (p<0.001) and with history of palatal fistula (p=0.032). Depth of nasopharynx was significantly greater in individuals with consistent hypernasality and nasal air emission (p<0.001). Depthlength ratio was significantly larger in individuals with consistent hypernasality and nasal air emission (p<0.001). A depth:length ratio larger than 0.93 was always associated with speech signs of VPD.

**Conclusion:**

Estimated with cephalometric radiographs, a depth:length ratio greater than 0.93, between the nasopharyngeal space and the velum, was 100% accurate in predicting hypernasality and nasal air emission after primary repair of unilateral cleft lip and palate.

## Introduction

During times of increasingly recognized importance of interprofessional practices, the interdisciplinary care of craniofacial anomalies is essential to improve quality of life and to reduce burden of care for patients and families. Professionals in Medicine, Dentistry, and Speech Pathology areas of healthcare interact to prevent and optimize the treatment of velopharyngeal dysfunction (VPD) and palatal fistula after primary palatoplasty. Not all cleft palate team worldwide, however, have access to imaging assessment of velopharyngeal function, such as videofluoroscopy or nasoendoscopy.^1^ Fayyaz, et al.^1^ (2019), for example, proposed assessing velopharyngeal competency as part of a “*system of classification for defining and describing palatal fistulae”*. According to the authors^1^, conducting a videofluoroscopic or nasoendoscopic evaluation “*would have been a better approach*”, but it is not always available, leading the authors to propose a clinical judgment regarding velopharyngeal competency, based in the intraoral examination combined to the outcome of speech assessment.

Although some cleft palate teams may lack equipment for videofluoroscopy or nasoendoscopy, most institutions have access or partnerships that deliver the cephalometric radiographs required during orthodontic follow-up to monitor growth, position, and size of skeletal and dental structures — as proposed in the *Parameters for Evaluation and Treatment of Patients with Cleft Lip/Palate or Other Craniofacial Differences.*^2^ If parameters from the American Cleft Palate-Craniofacial Association (ACPA) are followed, typical orthodontic evaluation should include cephalometric radiographs for all patients with cleft lip and palate (CLP). Measures of velopharyngeal structures, such as velar length (VL), velar thickness (VT) and depth of the nasopharynx (DN), can be obtained, routinely, for all patients (with and without signs of VPD), using cephalometric radiographs.

Using cephalometric radiographs to study the relationship between the velum and the pharynx is not a novel idea,^5,6,7,8,9,10^ but the literature still is not consistent regarding the predictive value of VL, VT, and DN for the management of VPD. Mazaheri, Athanasiou and Long^7^(1994) used cephalometric radiographs to compare VL, VT and DN between groups of patients with different types of CLP, with and without velopharyngeal competence for speech. The authors studied 85 individuals with cleft lip and palate, at “*6 month intervals during the first 2 postnatal years and annually thereafter up to 6 years of age”*, and reported that the measurements obtained indicated that it would be impossible to predict those individuals who would later require management of VPD. More recently, Silva, et al.^11^(2017), described size of velopharyngeal structures in individuals with VPD after primary palatal repair of cleft lip and palate. These later authors measured 90 still single-frame-videofluoroscopic images (similar to cephalometric radiographs) and reported that the presence of VPD could be predicted by the depth:length ratio measure in 71 out of 90 individuals studied.

The conflicting interpretation regarding the value of cephalometric measurements between studies^7,11^certainly warrant further investigation to verify the relationship between the velum and nasopharynx in individuals at risk of VPD, particularly when considering the potential use of pre-existing cephalometric radiographs obtained regularly by the dental team. Our study aims to compare velar length, velar thickness, and depth of the nasopharynx of patients with unilateral cleft lip and palate (UCLP), with the presence, or absence, of hypernasality and nasal air emission; and to verify if the depth:length ratio between nasopharynx and velum would be predictive of consistent hypernasality and nasal air emission (speech signs of VPD). Considering that velopharyngeal dysfunction is one of the most common complications of primary palatoplasty, researchers have been searching for pre-surgical anatomical evidences able to predict the success (or lack) of primary palatal repair. This could improve treatment protocols and avoid post-surgical complications. With improved cleft palate management protocols, it will be possible to reduce VPD, minimizing the stigma of hypernasal speech.

## Methodology

This study was approved by the institutional review board (#1.709.661). An available series of 466 cephalometric radiographs were analyzed for inclusion in this study. To meet the inclusion criteria, the radiographs had to be obtained from individuals with non-syndromic unilateral cleft lip and palate (UCLP), who were followed, consecutively, at a single research site. In addition, the cephalometric radiographs had to be taken before orthopedic and orthodontic management and prior to surgical management of velopharyngeal insufficiency. Routine settings for obtaining cephalometric radiographs to monitor growth, position, and size of skeletal and dental structures were followed during acquisition of the images selected. Exclusion criteria included cephalometric radiographs that were not adequately obtained, for example: images in which the velum was not at a physiological rest, the teeth were not in occlusion, or the head was not positioned adequately. Images in which the object of interest for our study were not identifiable were also excluded.

Each radiograph was analyzed by a single speech-language pathologist using the Dolphin Imaging Software (version 11.0). VL, VT and DN were measured using cephalometric procedures^12^, adjusted according to Subtelny^4^(1957) and Williams, Henningsson and Pegoraro-Krook^3^ (2004). The identification of the anterior nasal spine (ANS) and the posterior nasal spine (PNS) were indicated as the first steps for establishing VL, VT and DN by Williams, Henningsson and Pegoraro-Krook^3^ (2004), followed by the definition of the palatal plane. According to the authors,^3^ the palatal plane is established as “*a line drawn from the anterior nasal spine (ANS) through the posterior nasal spine (PNS) and extending back through the posterior pharyngeal wall (PPW)*”. The authors, however, reported difficulties in establishing a precise location for ANS and PNS in patients with operated cleft palate, suggesting the use of landmarks as the “*distal margin of the third molar and the inferior shadow of the pterygomaxillary fissure*” as a strategy for locating the most posterior margin of the hard palate.

In children between 6 and 9 years of age (participants of this study), the third molar is not available to be used as a reference, but third molar germs, when present, may be used as reference. In our study, both third molar germs (when present) and the retro molar maxillary tuber area were used as reference to identify PNS. Since the maxillary tuber is usually aligned with the anterior surface of the pterygomaxillary fissure, these landmarks were also functional references to locate the PNS. The ANS, although shortened or rotated due to the unilateral cleft lip and palate, could be visualized in the sample studied. The depth of the nasopharynx was measured after establishing the palatal plane, drawing a line from the anterior nasal spine (ANS) through the most posterior margin of the hard palate, and extending the line through the posterior pharyngeal wall (PPW). The distance between posterior margin of the hard palate and PPW indicated the nasopharyngeal depth. When the adenoid pad was present, the DN was measured between the posterior margin of the hard palate and the point where the palatal plane reached the adenoid.

The length of the soft palate (VL) was measured in its physiological rest position, from the most posterior margin of the hard palate to the tip of the uvula. The measure of velar thickness was established at the greatest thickness of the soft palate, and measured as the greatest distance from the dorsal to the ventral surfaces of the soft palate, drawn perpendicular to the line measuring the velar length. DN and VL were used to establish the depth:length ratio, by dividing the depth by the length.

History or presence of fistula formation and presence of hypernasality and nasal air emission were retrieved from patient’s charts. At the research site, the Test of Nasal Air Emission (TNAE) and the Test of Hypernasality (THYPER), adapted from Bzoch^13^ (2004), and described by Williams, et al.^14^ (2011) and by Pegoraro-Krook, Marino and Dutka^15^ (2019), are used, routinely, by speech-language pathologists for documenting speech outcome during management of cleft lip and palate. The THYPER^15^ compares the patient’s speech nasality with the nares closed (gently pinched by the clinician) to nasality with the nares open, during production of 10 oral consonant-vowel-consonant-vowel stimuli. This cul-de-sac test provides an index of the consistency of hypernasality ranging from 0 to 10, in which 0 indicates no perceptible difference in resonance between the open and closed nares conditions (interpreted as absence of hypernasality), and 10 indicating a perceptible shift occurring on each test word (interpreted as consistent hypernasality). The TNAE^15^ is performed using a mirror placed under the patient’s nose during the production of ten two-syllable words with oral pressure consonants. This test provides an index of the consistency of nasal air emission during oral speech; ranging from 0 to 10, in which 0 indicates no nasal air escape and 10 indicates nasal air escape on each test word. The speech-language pathologist that applied THYPER and TNAE had a minimum of 5 years of daily experience in perceptual assessment of speech of persons with CLP and VPD. Data from THYPER and TNAE were retrieved from patient’s charts, assuring that the dates of the speech assessment and the dates of the cephalometric radiographs were the same.

Data were analyzed using both, descriptive and inferential statistics. Means and standard deviations (SD) for VL, VT, DN and the depth:length ratio (DN/VL) were established, presented in Table 1 and compared. The hypothesis that participants with speech signs of VPD would present significantly different VL, VT, and DN measures than those without speech signs of VPD was tested using Student’s *t-*Test and Mann Whitney Test. The predictive value of the depth:length ratio (DN/VL) in estimating the presence of speech signs of VPD was established using the algorithm C4.5 (decision tree).

**Table 1:** Velar length (VL), velar thickness (VT), depth of nasopharynx (DN) and depth:length ratio (DN/VL ratio), grouped according to presence (WITH VPD) or absence of velopharyngeal dysfunction (WITHOUT VPD) and according to presence (WITH Fistula) or absence of palatal fistula (WITHOUT fistula)

VARIABLES	MEAN (SD) mm	INTERPRETATION OF FINDINGS
VL & WITHOUT VPD (N=307)	28.66 (3.57)	Mean VL was significantly shorter for those with VPD p<0.001)*
VL & WITH VPD (N=122)	26.47 (3.48)	
VT & WITHOUT VPD (N=307)	5.95 (1.55)	Mean VT was thinner for those with VPD (p=0.243)
VT & WITH signs of VPD (N=122)	5.69 (1.81)	
DN & WITHOUT VPD (N=307)	17.86 (4.30)	Mean DN was significantly greater for those with VPD (p<0.001)*
DN & WITH VPD (N=122)	19.74 (4.62)	
DN/VL ratio WITHOUT VPD (N=307)	0.63 (0.17)	Mean DN/VL ratio was significantly larger for those with VPD (p<0.001)*
DN/VL ratio WITH VPD (N=122)	0.76 (0.21)	
VL & WITHOUT fistula (N=361)	28.20 (3.73)	Mean VL was significantly shorter for the group with fistula (p=0.032)*
VL & WITH fistula (N=68)	27.17 (3.25)	
VT & WITHOUT fistula (N=361)	5.96 (1.58)	Mean VT was thinner for those with fistula (p=0.243)
VT & WITH fistula (N=68)	5.68 (1.23)	
DN & WITHOUT fistula (N=361)	18.36 (4.38)	Mean DN was greater for the group with fistula (p=0.914)
DN & WITH fistula (N=68)	18.60 (4.94)	
DN/VL ratio WITHOUT fistula (N=361)	0.66 (0.19)	Mean DN/VL ratio was larger for those with fistula (p=0.203)
DN/VL ratio WITH fistula (N=68)	0.69 (0.20)	

* p values indicating significant difference between groups

** VPD=velopharyngeal dysfunction (established as consistent hypernasality and nasal air emission)

VL: velar length

VT: velar thickness

DN: depth of nasopharynx

DN/VL: depth:length ratio

WITH VPD: presence of speech signs of velopharyngeal dysfunction

WITHOUT VPD: absence of speech signs of velopharyngeal dysfunction

WITH fistula: presence of palatal fistula after primary palatoplasty

WITHOUT fistula: absence of palatal fistula after primary palatoplasty

## Results

To evaluate the error of the method, we repeated the measurements 15 days later for 151 (35%) cephalometric radiographs randomly selected. We used the principles defined by Dahlberg (1940) to estimate the method error for each parameter.^16^ The differences between the first and the second measurements for VL, VT, DN were not significant with p=0.066, p=0.616, and p=0.806, respectively.

From the 466 radiographs available, we excluded 37 (8%) due to lack of quality of the image. In total, our study included 429 cephalometric radiographs from patients between 6 and 9 years of age (mean 7.2, SD 1.1). Within the group studied, 307 (72%) cephalometric radiographs belonged to patients without hypernasality and nasal air emission, whereas 122 (28%) belonged to patients with consistent hypernasality and nasal air emission as indicated by the THYPER and TNAE tests. At the time of data collection, the patients had not undergone surgical management of velopharyngeal insufficiency, but 68 (16%) individuals had undergone surgical repair of palatal fistula. That is, for 68 individuals, the speech evaluations and the cephalometric radiographs were documented after surgical correction of palatal fistula.

We established overall means and standard deviations (SD), as well as minimum and maximum values for VL, VT, DN and depth:length ratio. When considering velar length for all 429 individuals, the results indicated a mean of 28.04 mm (SD 3.68), a minimum value of 17.00 mm, and a maximum value of 39.70 mm. When considering velar thickness, the results indicated a mean measure of 5.91 mm (SD 1.53), a minimum value of 2.80 mm, and a maximum value of 11.40 mm. When considering depth of nasopharynx, the results indicated a mean measure of 18.40 mm (SD 4.47), a minimum value of 7.10 mm, and a maximum value of 31.20 mm. The depth:length ratio for the sample of 429 radiographs indicated a mean ratio of 0.67 (SD 0.19), a minimum ratio of 0.22, and a maximum ratio of 1.32. We regrouped data according to the presence or absence of speech signs of VPD, and according to the presence or absence of palatal fistula (Table 1).

Table 1 indicated that the mean velar length was significantly shorter for the group of 122 participants with speech signs of VPD (p<0.001) and was significantly shorter for the group of 68 patients with fistula (p=0.032). Whereas the mean depth of nasopharynx was significantly greater for the group of 122 participants with VPD (p<0.001)*, the mean depth of nasopharynx was greater for the group with fistula, but the difference was not statistically significant (p=0.914). Mean thickness was thinner for the group of 122 individuals with speech signs of VPD and for the 69 individuals with fistula, but the differences were not statistically significant (p=0.243 and p=0.243, respectively). Mean depth:length ratio was significantly larger for the group of 122 participants with VPD (p<0.001)* and was larger for the group of 68 participants with fistula, but not significant (p=0.203). A group of 35 individuals presented both, history of palatal fistula and hypernasality and nasal air emission. For these individuals, mean depth:length ratio, velar length, depth of nasopharynx and velar thickness were 0.74 (0.21), 26.22 (3.03), 19.18 (5.06), 5.41 (1.12), respectively.

When studying the predictive value of the depth:length ratio in estimating signs of VPD using the algorithm C4.5 (decision tree), we verified an association of a ratio larger than 0.93 with the presence of speech signs of VPD. When considering the group with fistula, we observed an association of a ratio larger than 0.79 with the presence of speech signs of VPD.

## Discussion

Knowledge about anatomical aspects of the structures of the velopharynx are rapidly expanding, involving the use of precise imaging of human body. The use of more advanced imaging methods, like Magnetic Resonance Imaging and Cone-Beam Computed Tomography Imaging, optimize the understanding of velopharyngeal dynamics and relationships between anatomical structures.^17,18,19,20^ However, many CLP teams around the world have no resources to perform even a nasoendoscopic or a videofluoroscopic assessment.^1^

While the use of 2-dimensional images with the velopharynx at rest — obtained with a cephalometric radiograph — may provide important information regarding the anatomical possibility of velopharyngeal closure, data obtained with 2D imaging may never substitute information derived from dynamic assessment of velopharyngeal function for speech. During the treatment decision process for management of velopharyngeal dysfunction (VPD), however, information obtainable with nasoendoscopic or videofluoroscopic assessments may be limited by the child’s compliance to the endoscope insertion and to exposure to larger amounts radiation, respectively. Despite being considered the gold standard tools for dynamic assessments of velopharyngeal function during speech^2^, the nasoendoscopic or the videofluoroscopic assessment is not routinely performed in individuals with normal speech after primary palatoplasty. Considering the widespread availability of still X-ray machines, the use of cephalometric radiographs can enhance both the diagnose of VPD and the study of velopharyngeal morphology due to its non-invasive nature and limited exposure to radiation.

Studies have described the characteristics of structures involved in velopharyngeal function using single lateral still X-rays or cephalometric radiographs.^4,7,8,9,10,21^ Subtelny^4^(1957), in particular, reported that a depth:length ratio greater than 0.70 suggested an unfavorable relationship between the velum and the pharynx, indicating that the DN/VL ratio could be used to predict and identify individuals at risk of VPD. Silva, et al.^11^(2017) reported that the sensitivity of the depth:length ratio as an index of VPD was 80%. Whereas participants in the study by Silva, et al.^11^ (2017) constituted a population diagnosed with speech signs of VPD and, therefore, were subjected to videofluoroscopic exams, our study presents data obtained with the use of cephalometric radiographs; extending the value of velar length (VL), velar thickness (VT), and depth of nasopharynx (DN) measurements also to the population with operated UCLP without speech signs of VPD. Our study identified that the presence of consistent hypernasality and nasal air emission could be predicted for all individuals (100%) with a depth:length ratio measure larger than 0.93.

Even when considering possible methodological differences in establishing VL, VT and DN among studies, the depth:length ratio allows the establishment of a relationship between the nasopharyngeal space and the velum. In this context, the greater the DN/VL ratio, the greater the estimated displacement required by the velum for achievement of closure. Considering Subtelny’s^4^ (1957) normative data for individuals without CLP, we verified that the author’s depth:length ratio measures ranged between 0.66 (SD 0.15) at 6 years, 0.70 (SD 0.14) at 7 years, 0.69 (SD 0.14) at 8 years, and 0.66 (SD 0.13) at 9 years. The mean depth:length ratio of 0.63 (SD 0.17) found in our study for the group without hypernasality and nasal air emission is similar to Subtelny’s data, and is predictive of possibility of velopharyngeal closure. That is, individuals without signs of VPD, in our study, had depth:length ratio measures within a range that corroborates their speech findings (absence of hypernasality and nasal air emission).

Considering the group with speech signs of VPD (N=56), nearly half (46%) presented depth:length ratio interpreted as *false negative* for presence of VPD (such as DN/VL ration at 0.70 or below). These findings can be partly explained by an enlarged adenoid tissue, which we ignored, and should be addressed in future research. While measures of VL, VT, DN and depth:length ratio can be particularly important in corroborating speech signs of VPD, in CLP Centers without access to the equipment required for nasoendoscopy and videofluoroscopy, the findings of 2D imaging require careful interpretation, considering the high incidence of false positives. Nevertheless, in our study, when the magnitude of the relationship between the nasopharyngeal space and the velum was at a ratio above 0.93, the DN/VL ratio predicted VPD with 100% accuracy.

Finally, one of the advantages of a cephalometric radiograph is its widespread availability, and likelihood of young children compliance. This suggests the possibility of obtaining a still lateral X-ray for speech assessment purposes even earlier than a dental cephalometric radiography. If this is the case, the use of a contrast of barium sulphate, applied to both nares and to the mouth, prior to obtaining the still X-ray/cephalometric radiograph for speech purpose, may help demarcate the structures, improving the identification of the oral and the nasal surfaces of the soft palate.

## Conclusion

A depth:length ratio between nasopharyngeal space and velum greater than 0.93, was 100% accurate in predicting hypernasality and nasal air emission after primary repair of unilateral cleft lip and palate. Velar length was significantly shorter in subjects with consistent hypernasality and nasal air emission (p<0.001) and with history of palatal fistula (p=0.032). Depth of nasopharynx was significantly greater in subjects with consistent hypernasality and nasal air emission (p<0.001). Depth:length ratio was significantly larger in subjects with consistent hypernasality and nasal air emission (p<0.001).
